# The Impact of Post-Harvest Potato Storage on (Deep-Fried) Potato Mash Properties

**DOI:** 10.3390/foods15081433

**Published:** 2026-04-20

**Authors:** Kathleen Hooyberghs, Stijn Reyniers, Paula Moldenaers, Ruth Cardinaels, Kristof Brijs, Jan A. Delcour

**Affiliations:** 1 Laboratory of Food Chemistry and Biochemistry, KU Leuven, Kasteelpark Arenberg 20, B-3001 Leuven, Belgiumkristof.brijs@kuleuven.be (K.B.); jan.delcour@kuleuven.be (J.A.D.); 2 Soft Matter, Rheology and Technology, Department of Chemical Engineering, KU Leuven, Celestijnenlaan 200F, Box 2424, B-3001 Leuven, Belgium; paula.moldenaers@kuleuven.be (P.M.); ruth.cardinaels@kuleuven.be (R.C.); 3 Processing and Performance of Materials, Department of Mechanical Engineering, TU Eindhoven, P.O. Box 513, 5600 MB Eindhoven, The Netherlands

**Keywords:** potato mashes, storage, deep frying, texture

## Abstract

This study investigated whether storage changes the composition of Fontane and Challenger potatoes, including their starch characteristics, and whether these changes impact the properties of (deep-fried) mashes made from these potatoes stored for up to eight months. Fontane mashes showed an increase in firmness and viscoelasticity when potatoes were stored for a longer time. Moreover, when deep-fried mashes were made with Fontane potatoes, more water evaporated during deep frying and the resultant oil content increased as a function of the storage duration of the potatoes used to make them. This was not observed in mashes made from Challenger potatoes. Since the potato composition, starch characteristics and molecular mobility in mashes were minimally impacted by potato storage for both cultivars, it is assumed that storage-induced changes in potato cell walls and/or pectin methyl esterase activity contribute to the observed differences between deep-fried mashes made from fresh versus stored Fontane potatoes. The acquired insights help understand how potato storage can impact the properties of (deep-fried) potato mash-based products and highlight the potential to mitigate storage-induced declines in product quality by selecting cultivars based on the potato storage time.

## 1. Introduction

Potatoes (*Solanum tuberosum* L.) are an important food crop, with a global production estimated at 375 million tons in 2022 [1]. Due to their broad adaptability to diverse climates [2], high yields and valuable nutritional composition, potatoes are essential for food security [3]. Potato tubers contain 63–87% water, 9–24% starch and minor portions (<5%) of protein, non-starch polysaccharides, lipids, free sugars and minerals [4,5]. In addition, potatoes contain water-soluble vitamins, more specifically 0.008 to 0.030% of fresh weight vitamin C and 0.0007 to 0.0037% of fresh weight B vitamins [6]. Yearly harvesting is limited to just a few months, although the industry needs a year-round supply in order to be able to have a continuous production process. It follows that long-term storage of potatoes is essential to guarantee the availability of raw materials and prevent factory downtime. During storage, potato quality can decline due to respiration, disease development, and dehydration. To minimize quality loss, temperature regulation, humidity control, and ventilation are used [7,8]. Moreover, sprout development and growth can be minimized by using sprout suppressants [9,10,11,12,13]. What happens during storage is not only impacted by the storage conditions but also depends on the cultivar [14] and the applied pre-harvest conditions [15].

The starch–sugar metabolism during potato storage is well understood [16,17,18,19,20,21,22,23,24]. At storage below 9 °C an imbalance between starch degradation and glycolysis can cause an accumulation of reducing sugars, which is often described as cold-induced sweetening or low-temperature sweetening [8,24,25]. This can be problematic as both glucose and fructose participate in Maillard reactions with free amino acids (especially asparagine) during deep frying [26]. Such reactions not only cause the end-product to be darker and more bitter, they also cause the formation of acrylamide, which is considered a carcinogen [27,28]. Moreover, the physicochemical characteristics of isolated starch may change in function during storage time [15,20,29].

Besides starch, potato cell wall material also undergoes changes during storage. For instance, potato pectin’s degree of methylation increases during the first three weeks of storage [30]. Moreover, potato pectin becomes more linear during storage. More specifically, longer chains of uronic acid residues are formed [30,31]. During storage, the activity of pectin methyl esterase also decreases [32]. Hence, it is no surprise that the texture of raw potato tissue changes during storage [30,32].

Post-harvest potato storage alters how potatoes respond to processing. For instance, it has been proposed that starch leakage during cooking is higher when potatoes are cooked after extended storage due to an increased fraction of starch molecules being smaller than the cell wall pores [33]. However, only minimal differences in the texture of cooked potatoes prepared from either fresh or stored raw material have been noted [31,32].

Significant quantities of potatoes are processed into a wide range of convenient frozen deep-fried food products such as croquettes, potato waffles, or *pommes duchesse*. These products typically contain potato mash as a base, making the potato mash properties critical for the quality of the end products. However, research on the impact of potato storage-induced changes on the properties of mashes, particularly when deep-fried, is currently lacking.

The storage-induced changes in raw potato composition (e.g., as a result of starch–sugar metabolism) are well documented and may translate into processed mash-based products in general and deep-fried items in particular. Given that starch, besides water, is the main component of potatoes, and is reported to undergo physicochemical modifications during storage, these alterations are hypothesized to impact the (deep-fried) potato mash properties. Such products are globally consumed and need to have a consistent texture and oil content. Additionally, how responses to storage depend on cultivar and how they impact mash production remains poorly understood and thus presents challenges for food manufacturers seeking to maintain product quality year round. Against this background, the aim of the present study was to investigate whether and, if so, to what extent and how potato storage-induced changes in potato composition and starch properties impact (deep-fried) potato mash properties.

## 2. Materials and Methods

### 2.1. Materials

Potatoes of the cultivars Fontane and Challenger, both of which are industry standards for mash-based products in Belgium, were harvested in September 2021 and subsequently stored in a dark, ventilated storage facility on a farm in Nallinnes (Belgium). Potatoes weighing between 100 and 250 g were sampled from the storage facility in September 2021, October 2021, November 2021, January 2022, February 2022, March 2022, April 2022 and May 2022 (only Fontane since Challenger was not available anymore). Prior to the analyses, they were stored in the dark at 6.5 °C for a maximum of 23 days. Risso Balance deep-frying oil was from Vandemoortele (Ghent, Belgium). Sodium acetate, fructose and ethanol were from VWR International (Leuven, Belgium). Lithium bromide (LiBr), rhamnose, sucrose, maltose, sodium bisulfite and isoamylase [*Pseudomonas* sp. (0–8124)] were from Sigma-Aldrich (Bornem, Belgium). Dimethyl sulfoxide, sodium hydroxide (NaOH), trifluoroacetic acid, HPLC grade acetic acid, glucose and sodium acetate (when used as a solvent for the sugar analysis) were from Thermo Scientific Chemicals (Waltham, MA, USA). Hexane (99+%) was from Chemlab (Zedelgem, Belgium).

### 2.2. Analyses of Fresh and Stored Potatoes

#### 2.2.1. Moisture Content

The moisture content (MC) of potato tissue (2.0 g) from the center and close to the edge of 10 potato tubers of each cultivar was determined gravimetrically in duplicate, following AACC International method 44-15-02 [34], which involves an overnight heating step at 130 °C.

#### 2.2.2. Weight Loss During Storage

At least 20 potatoes from each cultivar were stored separately at 6.5 °C in a dark room starting in October 2021 and their weight was monitored on a regular basis until sprouting started (February 2022).

#### 2.2.3. Composition

At the different storage times, 10 potatoes of each cultivar were peeled and freeze-dried. Moisture contents of accurately weighed freeze-dried potato tissue samples (1.0 g) were determined at least in duplicate as in Section 2.2.1 and used to express the compositions on a dry matter (DM) base. Protein contents (N × 6.25) were determined with an Elemental Analyzer 1108 (Carlo Erba Instruments, Milan, Italy).

The free sugars were extracted from 25 mg freeze-dried tissue after adding 9.990 mL MilliQ water and 10 µL internal standard (10.0 mg/mL rhamnose in MilliQ water) for 2 h while shaking (150 rpm). The suspensions were centrifuged at 4000× *g* for 20 min at 7 °C and the supernatants were filtered (0.22 µM; Merck-Millipore, Burlington, MA, USA). The samples were then analyzed by high-performance anion-exchange chromatography with pulsed amperometric detection (HPAEC-PAD) with a Thermo Fisher Scientific ICS-5000 HPAEC-PAD (MA, USA) device equipped with a PA-100 pre-column and a PA-100 analysis column. The mobile phase was 55 mM NaOH during column equilibration and the first 5 min of the run. Subsequently, the sodium acetate and NaOH concentrations in the mobile phase were increased from 0.0 to 4.8 mM and from 55.0 to 57.4 mM over 1.35 min and then from 4.8 to 91.2 mM and from 57.4 to 100.0 mM over 16.35 min. The flow rate was 1.00 mL/min. Standard calibration curves were constructed to identify and quantify rhamnose, sucrose, maltose, fructose and glucose. As the peak areas were below 1 nC·min for 6 of the 150 samples for fructose, they were considered too low to be precisely quantified, and the value was set at 0.5 nC·min.

To determine the total glucose content, 7.00 mg freeze-dried potato tissue was suspended in 250 µL MilliQ water and hydrolyzed with 500 µL 4.0 M trifluoroacetic acid for 1 h at 110 °C. Internal standard solution (250 µL 20.0 mg/mL rhamnose in MilliQ water) was added, the samples were diluted (1:500) and filtered (0.22 µm) prior to analysis with the above HPAEC-PAD device. The mobile phase was 100 mM NaOH during equilibration and the first 5 min of the run. Subsequently, the concentration of sodium acetate in the mobile phase was increased from 0.0 to 18.4 mM over 5 min, while keeping the NaOH concentration isocratic at 100 mM. The flow rate was 1.00 mL/min. The starch content was calculated by multiplying the total glucose content, after subtracting the free glucose content, by 0.9.

### 2.3. Isolation and Characterization of Starch

#### 2.3.1. Starch Isolation

Potato starch was isolated from 10 potato tubers of each cultivar at the different storage times [35]. Potatoes were peeled, washed, cut into pieces of approximately 2 to 3 cm and soaked for 30 min in demineralized water containing 0.04 g/L sodium bisulfite to inhibit browning. The total amount of raw material used thus varied with tuber size. Next, the cut potatoes were blended for 3 min with a food processor (Cook Expert XL, Magimix, Vincennes, France) into a slurry which was then manually sieved (250 µm). Demineralized water was added to the material that was retained on the sieve and the mixture was blended and sieved again. Demineralized water was added to the combined material that passed the sieve and the starch granules were allowed to settle for 2 h, after which the supernatant was discarded and demineralized water was added to the sediment. This was repeated four times, i.e., until the supernatant was transparent. Following centrifugation (15 min, 4 °C, 2000× *g*), the starch pellet was washed three times with ethanol. After a second centrifugation (15 min, 4 °C, 2000× *g*), the starch was gently dried for 48 h at 37 °C. Its purity was calculated as 0.9 times the glucose content obtained following hydrolysis, conversion to sorbitol acetate and triplicate analysis by gas chromatography as in [36].

#### 2.3.2. Granule Size Distribution

The particle size distribution of isolated starch was determined in a single replicate with a dynamic particle imaging apparatus (QicPic, Sympatec, Clausthal-Zellerfeld, Germany) equipped with a dry dispersion module (Oasisdry-Rodos, Sympatec) and an M5 lens, suitable for particles in the 1.8–1252 µm range. PAQXOS 6.1.2 software (Sympatec, Clausthal-Zellerfeld, Germany) was used to evaluate the size distribution by the diameter of their circle of equal projection area (EQPC) and to classify particles into 26 size classes. Only the particles with an EQPC smaller than 110 µm were considered, since larger particles are most likely agglomerates of starch granules. The denotations d10, d25, d50, d75 and d90 refer to the EQPC corresponding to 10, 25, 50, 75 and 90% of the cumulative frequency, respectively.

#### 2.3.3. Chain Length Distribution

High-performance size exclusion chromatography (HPSEC) was used to evaluate the chain length distribution of the isolated starches after debranching with isoamylase in a single replicate, performed essentially as in [37]. Molecule fragments were considered to have originated from amylopectin (AP) and amylose (AM) chains when their degrees of polymerization (DP) were between 10 and 110 or between 110 and 15,000, respectively. Based on the chain length distribution, the DP of AM and AP chains as well as the AM-to-AP ratio were calculated.

#### 2.3.4. Gelatinization Characteristics

Differential scanning calorimetry (DSC) was performed using a TA Instruments (New Castle, DE, USA) Q2000 DSC in a single replicate. Isolated starch samples (2.5–4.0 mg) were placed in PerkinElmer (Waltham, MA, USA) pans. Demineralized water was added at a 1:3 (*w*/*w*) DM to water ratio. The pans were hermetically sealed and equilibrated at 20 °C before heating from 20 to 120 °C at 4 °C/min. TA Universal Analysis software V4.0C was used to determine the gelatinization enthalpies (ΔHs) on starch basis and their associated temperatures.

#### 2.3.5. Pasting Properties

The swelling and disruption of the isolated starch granules during heating and their gel formation during cooling under shear were studied with a Rapid Visco Analyzer (RVA Super 4, Perten instruments, Hägersten, Sweden) in a single replicate. Accurately weighed samples (1.3–2.0 g DM) containing 1.25 g starch were suspended in demineralized water to obtain a starch concentration of 5.0% and a total weight of 25.0 g in an RVA cup. The viscosity of the suspension was monitored over time during a heating and cooling cycle under continuous stirring (160 rpm). Following an isothermal step (5 min at 50 °C), the temperature was increased at 9 °C/min to 95 °C, a second isothermal step was executed (8 min at 95 °C,) after which the temperature decreased at 9 °C/min until 50 °C, and a 5 min isothermal step at 50 °C was implemented. The viscosity was expressed in cP (i.e., mPa⋅s). Relevant parameters were the maximum viscosity reached during the heating phase, i.e., the peak viscosity; the minimum viscosity reached at the end of the holding phase at 95 °C, i.e., the minimum viscosity; the difference between the peak and minimum viscosity, i.e., the breakdown viscosity; the viscosity at the end of the experiment, i.e., the end viscosity; and the difference between the end and minimum viscosity, i.e., the setback viscosity.

### 2.4. Production of (Deep-Fried) Potato Mashes

Mashes were produced as in [37], with some adaptations. Manually peeled potatoes were cut into 1.0 × 1.0 cm^2^ beams of variable length. Beams (500 g, derived from 20 potatoes) were blanched in a 2 L Schott bottle containing 1400 mL tap water at 65 °C, which was placed in a shaking water bath (60 rpm) for 40 min at 65 °C. After blanching, the potatoes were steam-cooked for 45 min at 110 °C in a Magimix Cook Expert XL (Vincennes, France) Food Processor filled with 500 mL demineralized water. They were immediately mashed for 40 s at 633 rpm with the egg white beater of the food processor and sieved with the kitchen aid (Benton Harbor, MI, USA) 5KSMFGA food grinder attachment (fine grinding plate; 0.5 mm diameter) at speed 1. Nine potato mashes were made for each storage period and each cultivar and analyzed (see Section 2.5). Three mashes were used for MC and molecular mobility analysis. Three other mashes were sieved over a 250 µm sieve positioned in front of the fine grinding plate to remove chunks and ensure mash homogeneity before determining their viscous and elastic moduli. The final three mashes were used for firmness analysis. They were shaped into cylinders (55 mm length; 25 mm diameter) with a manual croquette press and deep-fried in a Casselin (Chaponnay, France) frying pan of 13 L in Risso Balance deep-frying oil (Vandemoortele, Gent, Belgium) that was periodically replaced at 180 °C for 75 s. After cooling to room temperature for about 30 min, their firmness and oil contents (OCs) were determined (see Section 2.6).

### 2.5. Analysis of Potato Mashes

#### 2.5.1. Moisture Content

The MC of 4.0 g accurately weighed potato mash was determined gravimetrically in triplicate after heating overnight at 130 °C as in Section 2.2.1. Since a moisture gradient exists in potato tubers, the MC of potato tissue from the center and the edge was measured.

#### 2.5.2. Firmness

The firmness (N/m^2^), i.e., the normal stress needed to compress the samples, was analyzed by compressing six mash cylinders (20 mm length, 21 mm diameter) to 25% of their original height at 1 mm/s after equilibration to room temperature for 60 min as in [37].

#### 2.5.3. Viscoelastic Properties

Mashes were passed through a Capitani (Como, Italy) pasta maker at 2.5 mm roll distance and positioned on the temperature (23 °C) controlled Peltier Plate of a TA Instruments HR20 rheometer. They were shaped into discs (40 mm diameter) with a stainless-steel cutting tool and then compressed with a parallel plate geometry with a crosshatched surface (40 mm diameter; TA instruments) until 5.0 N. The solvent trap was installed within the first 10 s of the 5 min relaxation time. The viscoelastic properties of mash were determined via a rotational rheometer by applying frequency (0.1% strain, 100 to 0.1 rad s^−1^ frequency) and amplitude (0.01 to 100% strain at 10 rad s^−1^) sweeps in a single replicate. The storage (G′) and loss (G″) moduli were calculated using TA Instruments Trios software V5.2.1. The loss tangent (tan **δ**) was calculated as the ratio of G″ to G′ at 1 rad/s. The slopes of linear regressions of log(G′) and log(G″) as a function of log (frequency) at 1 rad/s were calculated and are further referred to as n’ and n”, respectively. The critical strain, which indicates the end of the linear region, corresponded to a ~5% drop in G′ from the plateau of the amplitude sweep.

#### 2.5.4. Molecular Mobility

Time-domain proton nuclear magnetic resonance (TD ^1^H NMR) measurements of the proton distributions in potato mashes were executed with a Bruker (Rheinstetten, Germany) 0.47 T Minispec mq20 TD NMR with 20 MHz operating resonance frequency for 1H. Triplicate 300 mg potato mash samples were compressed in NMR tubes, which were then sealed and allowed to equilibrate at room temperature for at least 15 min before analysis in triplicate, with all measurements completed within 60 min. Analysis was at 25 ± 1 °C and spin–spin relaxation times (T2) were determined. Distributions of spin–spin relaxation times (T2) were analyzed using free induction decay (FID, 90° pulse, 2.86 μs) and Carr–Purcell–Meiboom–Gill (CPMG, 180° pulse, 5.42 μs, 0.1 ms tau delay) sequences. The FID and CPMG acquisition windows were 0.5 ms and 500 ms, collecting 500 and 2500 data points, respectively. The number of scans was 32 to enhance the signal-to-noise ratio with a 3.0 s recycle delay. The CONTIN algorithm [38] was used to transform T2 relaxation curves into continuous T2 distributions. These T2 distributions characterize molecular mobility, as they result from nuclear spins experiencing various immediate environments due to the samples’ compositional and structural heterogeneity [39]. FID areas were normalized to DM content, while CPMG areas were normalized to the sample mass. The full width at half maximum (FWHM) of the peaks was determined by fitting Gaussian functions to the peaks using the Microsoft Excel solver function to minimize the sum of squared errors between the data and the model after a first estimation of the amplitude, mean and width. This consistently resulted in a coefficient of determination (R2) exceeding 0.96. Since each proton population peak was fitted separately, the range of relaxation times was rather limited. As a result, the difference in sampling density within populations, caused by the exponential decay of the relaxation times, was also limited. This difference thus had a limited impact on the determined FWHM. Since magnetic field inhomogeneities contribute to the T2 relaxation of protons in more mobile environments detected by the FID, the proton fractions with T2 relaxation times exceeding 0.1 ms were excluded from the findings presented here. Peaks associated with the various proton relaxation time populations were characterized by (i) area (relative proton quantity), (ii) mean T2 relaxation time (molecular mobility), and (iii) FWHM (T2 distribution).

### 2.6. Analysis of Deep-Fried Potato Mashes

Six deep-fried samples were equilibrated at room temperature for about 60 min. Their outer parts were removed, and the firmness of the crust-free cylindrical middle sections (height 20 mm, diameter 15 mm) was analyzed as in Section 2.5.2. The crust and crust-free middle sections were accurately weighed to calculate the crust-to-filling ratio. The MCs of accurately weighed filling (about 2.0 g) or crust (about 1.0 g) samples were analyzed in triplicate as in Section 2.5.1. The mass and MC of the cylinders before and after deep frying allowed calculation of the extent of water evaporation during deep frying. The crusts and crust-free middle sections were flash frozen with liquid nitrogen, freeze-dried and their lipid contents were determined gravimetrically via three sequential extractions with hexane as in [39].

### 2.7. Statistical Analysis

The statistical analyses were conducted using JMP Pro 17 (SAS Institute, Cary, NC, USA). For each cultivar, significant differences were detected by performing one-way analysis of variation (ANOVA), with comparison of mean values using the Tukey multiple comparison test, after visual evaluation of the normal distribution of the data per storage time. The difference was considered significant when the *p*-value was smaller than 0.05.

Additionally, the effect of storage time on the measured variables was considered significant when the *p*-value of the regression analysis was smaller than 0.05. For each cultivar, the strength and direction of the linear relationship were evaluated using the corresponding Pearson correlation coefficient (R). R values close to 1 or −1 indicate that the data points closely follow a linear trend. The number of biological replicates is equal to the number of observations (n).

Challenger potatoes were only available until April 2022. However, removing the data from May 2022 from the Fontane dataset would result in the same conclusions. Therefore, this difference did not affect the comparability of results between cultivars.

## 3. Results and Discussion

### 3.1. Changes in Potato Composition During Storage

The MCs of potato tissue from the center and the edge of Fontane and Challenger potatoes are shown in Table 1. A decrease in MC of tissue from the potato center was observed in Fontane throughout the entire storage period (see Table 1) and in Challenger between one and seven months of storage (R = −0.46; *p* < 0.0001; n = 46). For tissue close to the outer edge of the potatoes, decreases were observed for Fontane but not for Challenger. Moreover, Fontane potatoes underwent a greater weight loss (7.2 ± 2.7%) compared with Challenger potatoes (4.2 ± 1.2%) after five months of storage at 6.5 °C in a dark room (see Section 2.2.2; *p* < 0001).

The starch, protein, sucrose, fructose and glucose contents of potato tubers are shown in Table 2. Although a decrease in starch content during potato storage has been noted previously [15,20,21], the starch content did not measurably change during storage in the present study. It may be that Fontane and Challenger cultivars have a relatively low susceptibility to starch degradation during storage at the present conditions. The sucrose content decreased during storage of Fontane and Challenger potatoes, while the fructose and glucose contents increased. The latter may be a result of cold-induced sweetening [8,23,25]. In addition, Challenger potatoes had high contents of sucrose, fructose and glucose after one month of storage, which then decreased after two months of storage. The maltose contents were too low to be quantified. The protein content remained constant during storage.

### 3.2. Changes in Starch Characteristics During Potato Storage

The d_10_, d_25_, d_50_, d_75_ and d_90_ values of the isolated starch granules, as well as the DPs of the starches’ AM and AP and their AM-to-AP ratios are shown in Table 3. Although all the measured percentiles of the particle size distribution increased significantly for the isolated starch granules of Fontane potatoes and the d_10_ and d_25_ increased for Challenger, the measured differences were limited. The granule architecture thus remained largely intact. Furthermore, the DP of AM and AP and the AM-to-AP ratio of isolated Fontane and Challenger starch did not change as a function of potato storage time (see Supplementary Table S1). That the molecular architecture of starch was not affected by potato storage likely resulted in the swelling behavior and gel strength to be consistent throughout the storage time.

The ΔHs, onset (T_o_), peak (T_p_) and conclusion (T_c_) temperatures as well as the gelatinization temperature ranges (T_c_–T_o_) of isolated starches, as determined via DSC, are shown in Table 4. Although T_o_ and T_p_ decreased significantly during storage for both Fontane and Challenger starches, this variation is considered negligible as the decrease was limited (by about 1 °C). The T_c_ and T_c_–T_o_ increased for Fontane starches. It may be that crystalline regions were slightly reorganized during storage, increasing the variability between granules. However, since ΔH did not change as a function of storage time, this indicates that the internal molecular order was preserved.

**Figure 1:**
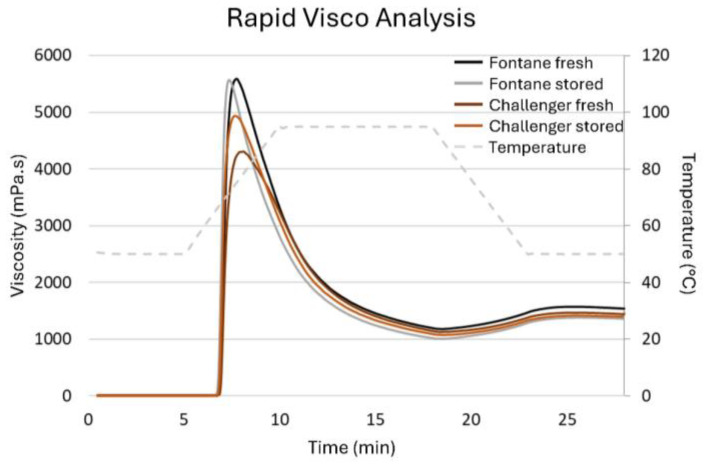
Rapid visco analyzer (RVA) profiles of 5.0% starch suspensions in demineralized water, prepared from starch isolated from potatoes that were either fresh or stored for 7 months (Challenger) or 8 months (Fontane).

Furthermore, the pasting properties of isolated starches are shown in Table 5 and Figure 1. While the pasting properties of Fontane starch remained unchanged in function during storage time, the peak, breakdown and setback viscosities increased for Challenger starch. In previous studies, starch thermal and pasting properties have been related to its granule size and chain length distribution [20,40]. Although the particle size distribution of the isolated starch granules changed only to a limited extent during storage (see Table 3), the increase in peak and breakdown viscosity correlated with the increasing percentiles of the particle size distribution for Challenger starch. However, these increases did not result in changed (deep-fried) potato mash properties (see Section 3.3 and Section 3.4), possibly because the end viscosity, which is a measure of amylose network formation, remained unaffected by potato storage.

Although there were some minor storage-induced changes in starch properties, the functional properties that affect the properties of (deep-fried) potato mashes were preserved. These minor changes thus likely did not translate into meaningful differences in (deep-fried) potato mash properties.

### 3.3. Changes in Mash Properties When Prepared from Potatoes Stored for Different Times

The MCs of mashes prepared from Fontane or Challenger potatoes as outlined in Section 2.4 ranged from 79.7 to 83.0% and from 78.6 to 83.5%, respectively (see Table 6). Although the MC range was limited, the MC of Fontane mashes showed a tendency to decrease when prepared from potatoes stored for increasing times, which corresponded with the MC data in Table 1 (see Section 3.1).

**Figure 2:**
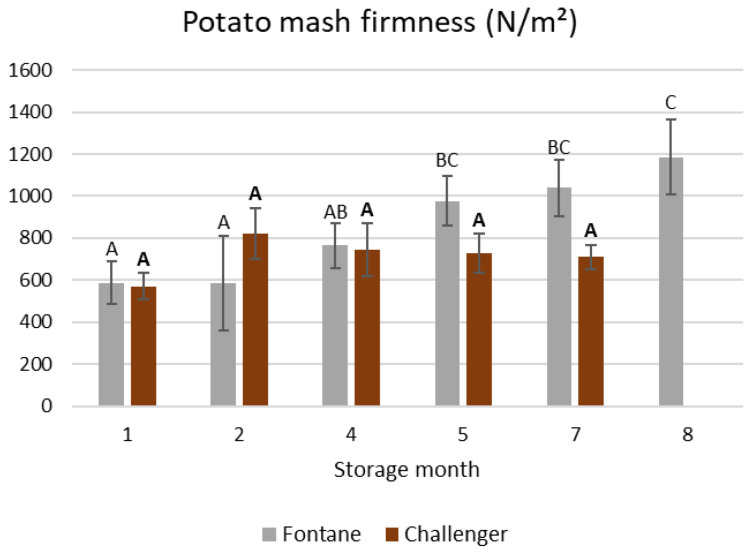
The firmness of mashes prepared from Fontane and Challenger potatoes stored for different times. Mean values differ significantly when they are assigned different Roman or bold letters for Fontane and Challenger, respectively. Error bars indicate the standard deviation.

**Figure 3:**
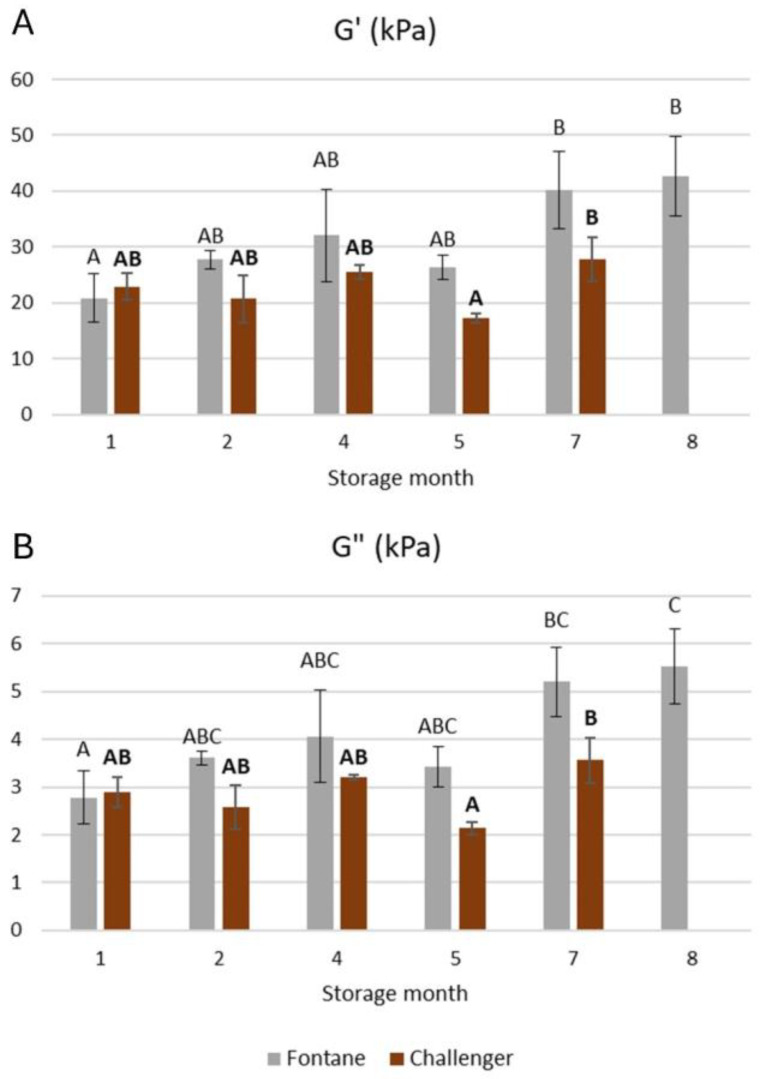
The storage modulus (G′; (**A**)) and loss modulus (G″; (**B**)) at 1 rad/s of mashes prepared from Fontane and Challenger potatoes stored for different times. Mean values differ significantly when they are assigned different Roman or bold letters for Fontane and Challenger, respectively. Error bars indicate the standard deviation.

The firmness of mash prepared from Fontane potatoes that were stored for eight months rather than for one month was 102% higher (see Figure 2). Similar differences in mash firmness have previously been observed when potato mashes were prepared with versus without a blanching step, which strongly influences potato mash properties [41]. In contrast, no such increase was observed for mashes made from Challenger potatoes. The spectra of G′ and G″ as a function of oscillatory frequency and strain for a representative mash sample are shown in Supplementary Figures S1A and S1B, respectively. The G′s and G″s are shown in Figure 3A,B, while the tan δs, n′s, n″s and critical strains are listed in Table 6. The G′ and G″ values and the critical strain remained constant for Challenger. In contrast, when mashes were prepared from Fontane potatoes stored for eight rather than one month, G′ and G″ were 126 and 110% higher, respectively, while the critical strain was 43% lower. Even if the limited decreases in the MC of Fontane mashes possibly resulted in slightly stiffer and less deformable mashes, other factors likely also influenced these characteristics. Indeed, given that the Challenger mashes had a similar MC range but did not show significant correlations between MC and firmness, G′ and G″, it can be concluded that, within this MC range, MC only has a small effect on these mash properties.

**Figure 4:**
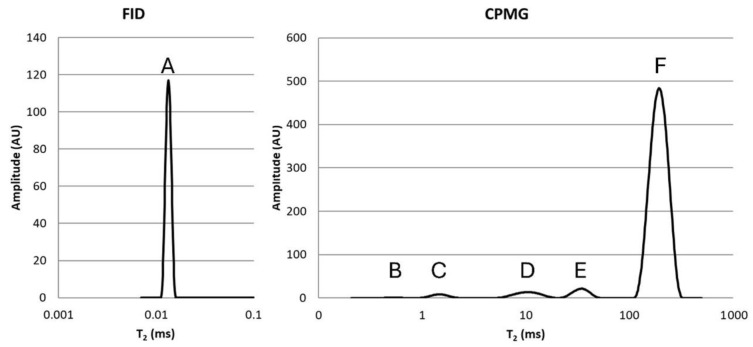
Proton distributions in a potato mash. The proton populations were obtained from free induction decay (FID) and Carr–Purcell–Meiboom–Gill (CPMG) sequences at 25 °C. Amplitudes are given in arbitrary units (AUs).

The distribution of proton T_2_ relaxation times in a typical potato mash sample is presented in Figure 4, with capital letters A to F indicating proton populations of increasing molecular mobility. The T_2_ relaxation time, area and FWHM of proton population A from the FID pulse sequence is shown in Table 7, while the data for the other two most abundant proton populations (‘E’ and ‘F’ from the CPMG pulse sequence) are shown in Supplementary Table S2. For both Fontane and Challenger mashes, the T_2_ relaxation time of population A, representing CH protons of crystalline or amorphous biopolymers in a rigid state not in contact with water [42], increased when the potato raw material had been stored for a longer time. However, the T_2_ relaxation time of this population ranged only over 1 µs and therefore this difference was considered negligible. Populations E and F represent protons of water closely associated with biopolymers and the exchangeable protons of the latter [43] and more mobile water protons associated with the gel structure [44,45], respectively. Since the impact of potato storage on the area and width of mobile proton populations E and F was also limited, the impact of potato storage on the molecular mobility of protons in potato mashes is here considered to be limited.

### 3.4. Changes in Deep-Fried Potato Mashes When Prepared from Potatoes Stored for Different Times

**Figure 5:**
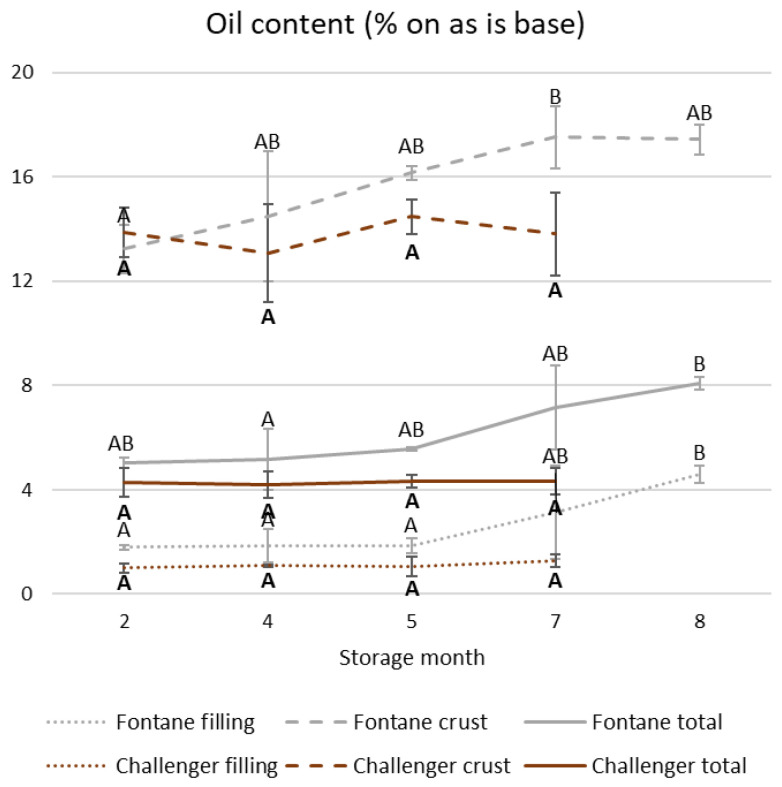
The filling, crust and total oil contents (OCs) of deep-fried potato mashes prepared from Fontane or Challenger potatoes stored for different times. Mean values differ significantly when they are assigned different Roman or bold letters for Fontane and Challenger, respectively. Error bars indicate the standard deviation.

The OCs of the fillings and the crusts and the resultant total OCs of deep-fried Fontane and Challenger potato mashes are shown in Figure 5 and Supplementary Table S3. Their corresponding crust-to-filling ratio and the water evaporation of deep-fried Fontane and Challenger potato mashes are shown in Table 8. These values did not depend on the storage time of the Challenger potatoes. In contrast, water evaporation during deep frying and the resultant OCs increased with longer prior storage of Fontane potatoes.

Although a lower mash MC would be expected to reduce the oil uptake as a result of deep frying, this was not observed for the Fontane mashes. Furthermore, the minimal or negligible changes in starch characteristics caused by potato storage (see Section 3.2) indicate that storage-induced changes in starch did not contribute to the increased OC in deep-fried mashes prepared from stored Fontane potatoes. Moreover, TD ^1^H NMR measurements of mashes prepared from potatoes stored for different times showed that the molecular mobility of water protons remained constant over the storage period (see Section 3.3). Hence, the observed increase in oil uptake as a result of deep frying of mashes prepared from longer-stored Fontane potatoes was not due to changes in molecular mobility of protons in potato mashes. It is probable that the microstructure present before and formed during deep frying allowed more water to evaporate and, consequently, more oil to absorb [46]. It is here hypothesized that potato storage increases the cell wall intactness and/or decreases the degree of cell clustering in mashes, which in turn impacts the presence of pores both before and during deep frying. Furthermore, the changes in cell wall intactness and degree of cell clustering possibly contributed to the increased firmness, G′ and G″ of mashes when the potato raw material had been stored for a longer time (see Section 3.3). Storage has been reported to enzymatically modify pectin composition and structure [31] and/or decreased pectin methyl esterase activity during blanching when potatoes have been stored for extended periods of time [32]. It may be that Fontane potatoes are more prone compared with Challenger to these enzymatic modifications during potato storage, which are here hypothesized to result in more pectin depolymerization during cooking of stored rather than fresh potatoes. The degree of cell separation and cell rupturing during mashing is hypothesized to be affected by pectin solubilization. Consequently, the ratio between intact cells, broken cells, cell clusters and extracellular material likely differs, affecting the (deep-fried) potato mash properties.

The firmness of the fillings increased with longer storage of the potato raw material for Fontane but not for Challenger potatoes, which is in line with the observations for potato mash firmness. The fact that the firmness and rheological properties of mashes, as well as the firmness and OC of deep-fried mashes, changed for Fontane but not for Challenger potatoes stored for different times highlights the cultivar dependence of storage-induced changes in potatoes and their impact on the quality of the end product.

Despite the current efforts to elucidate the impact of storage-induced changes on the properties of (deep-fried) potato mashes, further research remains warranted. For example, future studies can investigate different storage treatments and include more potato cultivars. Moreover, second, the scope of analysis of (deep-fried) potato mashes could be expanded. In addition to investigating the previously proposed hypothesis that pectin depolymerization is more pronounced during the cooking of stored compared with fresh potatoes, other aspects—such as sensory evaluation, as well as the stability of vitamins and frying oil—merit further attention.

## 4. Conclusions

While it is well established that potatoes undergo changes during long-term post-harvest storage, prior to the present work, it was unclear whether these changes affect (deep-fried) potato mash properties. In this study, the changes in raw potato composition, isolated starch characteristics and potato mash properties were collectively examined during storage.

The potato composition and the characteristics of starch isolated from Fontane and Challenger potatoes were minimally impacted by potato storage time. The potato composition and the starch characteristics could thus not explain why Fontane mashes became firmer and more solid-like over time. Moreover, water evaporation during deep frying and the resultant OC of deep-fried potato mashes increased when Fontane potatoes were used later. It is hypothesized that the storage-induced changes in (deep-fried) potato mash properties are due to alterations in potato cell wall composition and/or pectin methyl esterase activity, which results in increased pectin depolymerization during the production of mashes. The potential effects of these structural and enzymatic changes during storage on the properties of (deep-fried) potato mashes warrant further investigation.

Furthermore, that these changes were observed for Fontane but not for Challenger mashes indicates that Fontane potatoes are more prone compared with Challenger potatoes to these hypothesized enzymatic modifications during potato storage. Overall, this study highlights the potential to mitigate storage-induced declines in product quality by selecting potato cultivars based on the potato storage time.

## Figures and Tables

**Table 1:** The moisture content (MC) of tissue from the center and near the edge of Fontane and Challenger potatoes over the course of storage.

	Fontane		Challenger	
Storage Month	MC Center (%)	MC Edge (%)	MC Center (%)	MC Edge (%)
0	82.1 (1.3) ^A^	80.6 (1.2) ^A^	76.2 (3.6) ^A^	75.6 (4.1) ^A^
1	82.3 (1.0) ^A^	80.0 (0.7) ^A^	79.1 (1.7) ^A^	77.2 (2.0) ^A^
2	78.5 (2.2) ^B^	77.1 (2.3) ^BC^	79.1 (1.7) ^A^	77.1 (2.1) ^A^
4	78.1 (2.2) ^B^	78.3 (2.5) ^ABC^	78.2 (1.5) ^A^	76.9 (1.7) ^A^
5	82.6 (0.9) ^A^	79.7 (0.8) ^AB^	77.6 (1.7) ^A^	77.4 (2.3) ^A^
7	76.6 (1.7) ^BC^	75.9 (1.3) ^C^	76.8 (2.5) ^A^	75.6 (2.8) ^A^
8	76.5 (3.4) ^C^	75.3 (2.9) ^C^		
*p*-value	<0.0001	<0.0001	0.5230	0.7761
R	−0.61	−0.48	−0.09	−0.04
n	70	70	56	56

Standard deviations are given between brackets. For each cultivar, mean values in the same column differ significantly (*p* < 0.05, Tukey’s test) when they show different letters. The *p-*values, corresponding Pearson correlation coefficients (R) and number of observations (n) of the regression analysis are also reported.

**Table 2:** The starch, protein, sucrose, fructose and glucose contents of Fontane and Challenger potatoes over the course of storage expressed on dry matter (DM) basis.

	**Fontane Composition (% of DM)**				
**Storage Month**	**Starch**	**Protein**	**Sucrose**	**Fructose**	**Glucose**
0	67.0 (4.6) ^AB^	8.4 (3.6) ^A^	0.81 (0.20) ^A^	0.05 (0.02) ^A^	0.09 (0.04) ^A^
1	67.6 (2.2) ^AB^	7.3 (2.1) ^A^	0.53 (0.10) ^B^	0.08 (0.07) ^AB^	0.19 (0.10) ^AB^
2	70.6 (3.8) ^A^	6.8 (1.3) ^A^	0.47 (0.08) ^BC^	0.06 (0.03) ^A^	0.18 (0.07) ^AB^
4	70.4 (4.6) ^A^	7.7 (1.7) ^A^	0.41 (0.11) ^BC^	0.08 (0.04) ^AB^	0.19 (0.08) ^AB^
5	64.9 (2.6) ^B^	7.8 (2.0) ^A^	0.36 (0.10) ^BC^	0.35 (0.30) ^C^	0.42 (0.32) ^BCD^
6	68.8 (3.3) ^AB^	7.4 (2.1) ^A^	0.49 (0.18) ^B^	0.55 (0.21) ^C^	0.63 (0.23) ^CD^
7	70.6 (2.2) ^A^	6.6 (1.9) ^A^	0.41 (0.17) ^BC^	0.56 (0.29) ^C^	0.71 (0.46) ^D^
8	70.8 (2.6) ^A^	7.3 (1.6) ^A^	0.28 (0.09) ^C^	0.32 (0.12) ^BC^	0.39 (0.15) ^ABC^
*p*-value	0.0795	0.3363	<0.0001	<0.0001	<0.0001
R	0.20	−0.11	−0.60	0.63	0.57
n	80	80	80	80	80
	**Challenger Composition (% of DM)**				
**Storage Month**	**Starch**	**Protein**	**Sucrose**	**Fructose**	**Glucose**
0	67.3 (5.2) ^A^	7.6 (2.0) ^A^	0.78 (0.29) ^A^	0.03 (0.02) ^A^	0.08 (0.03) ^A^
1	65.6 (3.9) ^A^	7.9 (1.3) ^A^	1.44 (0.60) ^B^	0.53 (0.34) ^B^	0.64 (0.38) ^BC^
2	70.0 (2.2) ^A^	8.0 (1.4) ^A^	0.60 (0.19) ^A^	0.15 (0.11) ^A^	0.25 (0.17) ^AB^
4	67.8 (4.4) ^A^	8.0 (1.7) ^A^	0.61 (0.40) ^A^	0.16 (0.07) ^A^	0.27 (0.25) ^AB^
5	67.8 (3.5) ^A^	7.7 (1.9) ^A^	0.37 (0.10) ^A^	0.24 (0.08) ^A^	0.34 (0.14) ^ABC^
6	67.7 (3.3) ^A^	7.5 (1.0) ^A^	0.43 (0.08) ^A^	0.21 (0.09) ^A^	0.25 (0.11) ^AB^
7	70.2 (7.7) ^A^	6.8 (1.0) ^A^	0.43 (0.16) ^A^	0.55 (0.36) ^B^	0.74 (0.62) ^C^
*p*-value	0.2089	0.3905	<0.0001	0.0410	0.0560
R	0.15	−0.10	−0.53	0.24	0.23
n	70	70	70	70	70

Standard deviations are given between brackets. For each cultivar, mean values in the same column differ significantly (*p* < 0.05, Tukey’s test) when they show different letters. The *p-*values, corresponding Pearson correlation coefficients (R) and number of observations (n) of the regression analysis are also reported.

**Table 3:** The particle diameters of isolated starch granules from Fontane and Challenger potatoes, expressed as the diameter of a circle of equal projection area (EQPC) corresponding to 10, 25, 50, 75 and 90% of the cumulative frequency (d_10_, d_25_, d_50_, d_75_ and d_90_, respectively) as a function of potato storage time.

**Fontane**					
**Storage Month**	**d_10_ (µm)**	**d_25_ (µm)**	**d_50_ (µm)**	**d_75_ (µm)**	**d_90_ (µm)**
0	21.2 (1.9) ^AB^	28.4 (2.4) ^AB^	38.1 (3.3) ^AB^	46.9 (4.0) ^AB^	54.4 (3.8) ^AB^
1	20.8 (1.1) ^AB^	28.1 (1.6) ^AB^	38.1 (1.8) ^AB^	47.7 (2.4) ^AB^	55.3 (2.6) ^AB^
2	20.4 (1.4) ^B^	27.2 (1.6) ^B^	36.4 (1.9) ^B^	45.4 (2.4) ^B^	52.8 (3.0) ^B^
4	21.8 (0.9) ^AB^	29.2 (1.2) ^AB^	39.2 (1.5) ^AB^	48.9 (2.3) ^AB^	56.6 (2.7) ^AB^
5	21.9 (1.3) ^AB^	29.5 (2.2) ^AB^	39.4 (2.8) ^AB^	48.7 (3.5) ^AB^	56.4 (3.8) ^AB^
6	22.4 (1.4) ^A^	29.8 (2.1) ^AB^	39.6 (2.9) ^AB^	48.9 (3.4) ^AB^	56.5 (3.5) ^AB^
7	22.6 (1.1) ^A^	30.1 (1.4) ^A^	40.2 (1.7) ^A^	50.1 (2.4) ^A^	58.1 (2.7) ^A^
8	21.7 (1.6) ^AB^	28.9 (2.1) ^AB^	38.9 (2.8) ^AB^	48.9 (3.8) ^AB^	56.8 (4.4) ^AB^
*p*-value	0.0013	0.0037	0.0073	0.0034	0.0021
R	0.35	0.32	0.30	0.32	0.34
n	80	80	80	80	80
**Challenger**					
**Storage Month**	**d_10_ (µm)**	**d_25_ (µm)**	**d_50_ (µm)**	**d_75_ (µm)**	**d_90_ (µm)**
0	22.3 (1.8) ^AB^	30.5 (2.3) ^AB^	40.6 (2.8) ^AB^	50.5 (3.7) ^A^	58.8 (4.1) ^A^
1	21.7 (1.3) ^AB^	29.0 (1.7) ^AB^	38.0 (2.3) ^AB^	47.0 (2.7) ^AB^	54.7 (3.0) ^AB^
2	21.1 (2.1) ^B^	27.9 (2.5) ^B^	36.6 (2.8) ^B^	45.3 (3.2) ^B^	52.9 (3.4) ^B^
4	24.0 (1.7) ^A^	32.2 (2.9) ^A^	41.7 (3.0) ^A^	51.1 (2.7) ^A^	59.0 (2.3) ^A^
5	23.0 (1.9) ^AB^	30.8 (2.4) ^AB^	40.0 (2.8) ^AB^	48.9 (3.2) ^AB^	56.6 (3.4) ^AB^
6	23.0 (1.8) ^AB^	30.5 (2.9) ^AB^	39.5 (3.6) ^AB^	48.5 (4.1) ^AB^	56.4 (4.4) ^AB^
7	23.8 (2.6) ^A^	31.8 (3.2) ^A^	41.1 (3.9) ^A^	50.1 (4.9) ^AB^	57.7 (5.7) ^AB^
*p*-value	0.0045	0.0194	0.0825	0.2766	0.3918
R	0.34	0.28	0.21	0.13	0.10
n	70	70	70	70	70

Standard deviations are given between brackets. For each cultivar, mean values in the same column differ significantly (*p* < 0.05, Tukey’s test) when they show different letters. The *p*-values, corresponding Pearson correlation coefficients (R) and number of observations (n) of the regression analysis are also reported.

**Table 4:** Gelatinization enthalpy (ΔH), onset (T_o_), peak (T_p_) and conclusion (T_c_) temperatures and the temperature range of gelatinization (T_c_–T_o_) of starch isolated from potatoes at different storage time points.

**Fontane**					
**Storage Month**	**Δ** **H (J/g Starch DM)**	**T_0_ (°C)**	**T_p_ (°C)**	**T_C_ (°C)**	**T_c_-T_o_ (°C)**
0	25.9 (1.1) ^ABC^	60.4 (1.0) ^A^	62.8 (1.2) ^A^	67.6 (1.6) ^A^	7.2 (0.8) ^A^
1	22.0 (1.4) ^A^	60.2 (0.4) ^AB^	62.7 (0.5) ^A^	68.0 (1.2) ^AB^	7.9 (1.1) ^A^
2	29.4 (4.9) ^D^	59.4 (0.6) ^B^	61.8 (0.6) ^A^	70.5 (3.0) ^BC^	11.1 (3.1) ^B^
4	24.8 (0.9) ^ABC^	59.5 (0.5) ^AB^	62.3 (0.6) ^A^	71.5 (1.4) ^C^	12.0 (1.1) ^B^
5	23.0 (1.3) ^AB^	59.5 (0.8) ^AB^	62.2 (1.1) ^A^	67.5 (2.8) ^A^	8.1 (2.1) ^A^
6	26.3 (2.0) ^BCD^	59.4 (0.9) ^AB^	61.9 (1.1) ^A^	70.6 (1.9) ^BC^	11.2 (1.3) ^B^
7	27.5 (3.9) ^CD^	59.6 (0.7) ^AB^	62.2 (0.8) ^A^	71.7 (1.1) ^C^	12.1 (0.6) ^B^
8	25.8 (2.2) ^BCD^	59.3 (0.5) ^B^	61.9 (0.6) ^A^	71.6 (0.9) ^C^	12.2 (0.6) ^B^
*p*-value	0.3540	0.0014	0.0228	<0.0001	<0.0001
R	0.10	−0.35	−0.25	0.46	0.57
n	80	80	80	80	80
**Challenger**					
**Storage Month**	**Δ** **H (J/g Starch DM)**	**T_0_ (°C)**	**T_p_ (°C)**	**T_C_ (°C)**	**T_c_-T_o_ (°C)**
0	24.6 (1.7) ^A^	60.6 (0.4) ^A^	62.8 (0.4) ^A^	71.1 (1.6) ^A^	10.5 (1.4) ^AB^
1	24.9 (0.6) ^A^	60.1 (0.5) ^AB^	62.3 (0.5) ^ABC^	71.6 (0.7) ^A^	11.5 (0.7) ^A^
2	24.5 (2.5) ^A^	60.3 (0.6) ^AB^	62.6 (0.7) ^AB^	71.8 (1.6) ^A^	11.5 (1.1) ^A^
4	26.2 (2.6) ^A^	59.4 (0.3) ^C^	61.7 (0.3) ^C^	70.9 (1.0) ^A^	11.5 (0.8) ^A^
5	25.0 (2.7) ^A^	60.2 (0.3) ^AB^	62.5 (0.3) ^AB^	69.1 (1.6) ^A^	9.3 (2.4) ^B^
6	24.9 (2.1) ^A^	59.9 (0.3) ^BC^	62.1 (0.4) ^BC^	69.9 (1.0) ^A^	10.0 (0.9) ^AB^
7	26.3 (2.3) ^A^	59.8 (0.6) ^BC^	61.9 (0.7) ^C^	70.5 (1.6) ^A^	10.8 (1.5) ^AB^
*p*-value	0.0726	0.0003	0.0159	0.0041	0.0594
R	0.22	−0.42	−0.29	−0.34	−0.23
n	70	70	70	70	70

Standard deviations are given between brackets. For each cultivar, mean values in the same column differ significantly (*p* < 0.05, Tukey’s test) when they show different letters. The *p-*values, corresponding Pearson correlation coefficients (R) and number of observations (n) of the regression analysis are also reported.

**Table 5:** The peak (PV), breakdown (BDV), minimum (MV), setback (SBV) and end (EV) viscosities of 5.0% starch dry matter (DM) suspensions of starch isolated from potatoes at different storage time points.

**Fontane**					
**Storage Month**	**PV (mPa.s)**	**BDV (mPa.s)**	**MV (mPa.s)**	**SBV (mPa.s)**	**EV (mPa.s)**
0	5694 (717) ^ABC^	4511 (608) ^A^	1183 (151) ^AB^	362 (24) ^AB^	1545 (169) ^AB^
1	5049 (636) ^A^	3915 (610) ^A^	1134 (123) ^ABC^	350 (16) ^B^	1484 (134) ^AB^
2	6818 (2121) ^BC^	5587 (1939) ^A^	1231 (221) ^ABC^	445 (146) ^B^	1676 (354) ^AB^
4	5025 (707) ^A^	3901 (629) ^A^	1124 (112) ^ABC^	344 (22) ^B^	1468 (129) ^AB^
5	5232 (825) ^AB^	4040 (801) ^A^	1192 (76) ^AB^	363 (19) ^AB^	1555 (80) ^AB^
6	6171 (1336) ^ABC^	5039 (1188) ^A^	1132 (156) ^BC^	464 (128) ^A^	1595 (231) ^AB^
7	7133 (1454) ^C^	5881 (1336) ^B^	1252 (163) ^A^	426 (94) ^AB^	1678 (226) ^A^
8	5942 (43) ^ABC^	4902 (37) ^AB^	1041 (8) ^C^	373 (9) ^AB^	1366 (15) ^B^
*p*-value	0.1076	0.0615	0.3028	0.1922	0.8436
R	0.18	0.21	−0.12	0.15	−0.02
n	80	80	80	80	80
**Challenger**					
**Storage Month**	**PV (mPa.s)**	**BDV (mPa.s)**	**MV (mPa.s)**	**SBV (mPa.s)**	**EV (mPa.s)**
0	4579 (708) ^ABC^	3457 (718) ^ABCD^	1122 (91) ^A^	318 (19) ^AB^	1440 (107) ^A^
1	3976 (411) ^AB^	2969 (404) ^ABC^	1007 (29) ^A^	293 (10) ^A^	1301 (32) ^A^
2	3630 (467) ^A^	2543 (429) ^A^	1088 (130) ^A^	305 (13) ^AB^	1393 (135) ^A^
4	4648 (498) ^BC^	3605 (528) ^BCD^	1044 (73) ^A^	318 (14) ^AB^	1362 (75) ^A^
5	3884 (748) ^AB^	2786 (720) ^AB^	1098 (84) ^A^	326 (31) ^AB^	1424 (106) ^A^
6	4796 (1212) ^BC^	3809 (1146) ^CD^	987 (83) ^A^	310 (47) ^AB^	1298 (126) ^A^
7	5349 (837) ^C^	4234 (785) ^D^	1116 (156) ^A^	342 (67) ^B^	1458 (218) ^A^
*p*-value	0.0038	0.0027	0.7597	0.0323	0.7471
R	0.34	0.35	−0.04	0.26	0.04
n	70	70	70	70	70

Standard deviations are given between brackets. For each cultivar, mean values in the same column differ significantly (*p* < 0.05, Tukey’s test) when they show different letters. The *p-*values, corresponding Pearson correlation coefficients (R) and number of observations (n) of the regression analysis are also reported.

**Table 6:** The moisture content (MC), loss tangent (tan δ), slopes of linear regressions of log(G′) and log(G″) as a function of log(frequency) (n’ and n”, respectively) at 1 rad/s and critical strain of mashes prepared from Fontane and Challenger potatoes stored for different times.

**Fontane**					
**Storage Month**	**MC (%)**	**Tan** **δ**	**n′**	**n″**	**Critical Strain (%)**
1	82.4 (0.5) ^A^	0.13 (0.00) ^A^	0.07 (0.00) ^A^	0.03 (0.02) ^A^	0.26 (0.05) ^A^
2	81.6 (0.4) ^A^	0.13 (0.00) ^A^	0.07 (0.00) ^A^	0.04 (0.03) ^A^	0.19 (0.01) ^A^
4	82.4 (0.5) ^A^	0.13 (0.00) ^A^	0.06 (0.00) ^A^	0.03 (0.01) ^A^	0.22 (0.06) ^A^
5	81.8 (0.5) ^A^	0.13 (0.01) ^A^	0.07 (0.01) ^A^	0.05 (0.00) ^A^	0.20 (0.01) ^A^
7	81.4 (1.6) ^A^	0.13 (0.00) ^A^	0.07 (0.00) ^A^	0.01 (0.01) ^A^	0.15 (0.03) ^A^
8	80.9 (0.7) ^A^	0.13 (0.00) ^A^	0.07 (0.00) ^A^	0.01 (0.02) ^A^	0.15 (0.02) ^A^
*p*-value	0.0510	0.2632	0.6535	0.0773	0.0026
R	−0.47	−0.30	−0.12	−0.45	−0.70
n	18	16	16	16	16
**Challenger**					
**Storage Month**	**MC (%)**	**Tan** **δ**	**n′**	**n″**	**Critical Strain (%)**
1	83.2 (0.2) ^A^	0.13 (0.00) ^A^	0.07 (0.00) ^A^	0.01 (0.00) ^A^	0.25 (0.02) ^A^
2	81.8 (0.7) ^A^	0.12 (0.00) ^A^	0.07 (0.01) ^A^	0.03 (0.01) ^B^	0.30 (0.04) ^A^
4	82.5 (0.6) ^A^	0.13 (-) ^A^	0.07 (-) ^A^	0.01 (-) ^A^	0.26 (-) ^A^
5	82.2 (0.6) ^A^	0.12 (0.00) ^A^	0.07 (0.00) ^A^	0.03 (0.00) ^A^	0.30 (0.07) ^A^
7	82.3 (1.3) ^A^	0.13 (0.00) ^A^	0.06 (0.00) ^A^	0.02 (0.01) ^A^	0.21 (0.02) ^A^
*p*-value	0.6139	0.4209	0.4100	0.8208	0.1914
R	0.15	0.24	−0.25	0.07	−0.39
n	14	13	13	13	13

Standard deviations are given between brackets and greater than zero in all cases. For each cultivar, mean values in the same column differ significantly (*p* < 0.05, Tukey’s test) when they show different letters. The *p-*values, corresponding Pearson correlation coefficients (R) and number of observations (n) of the regression analysis are also reported.

**Table 7:** The area, T_2_ relaxation time (T_2_) and full width at half maximum (FWHM) of proton populations A, E and F in potato mashes prepared from Fontane and Challenger potatoes stored for different times.

**Storage Month**	**T_2_A (µs)**	**Area A/g DM (au)**	**FWHM A (µs)**
2	12.9 (0.0) ^AB^	11,722 (1948) ^A^	2.2 (0.1) ^A^
4	13.1 (0.2) ^ABC^	12,978 (479) ^A^	2.1 (0.2) ^A^
5	12.9 (0.2) ^A^	12,978 (749) ^A^	2.3 (0.5) ^A^
7	13.5 (0.2) ^C^	12,536 (963) ^A^	2.0 (0.2) ^A^
8	13.4 (0.1) ^BC^	12,182 (714) ^A^	2.0 (0.3) ^A^
*p*-value	0.0039	0.9586	0.4150
R	0.72	0.02	−0.24
n	14	14	14
**Storage Month**	**T_2_A (µs)**	**Area A/g DM (au)**	**FWHM A (µs)**
2	13.1 (0.1) ^A^	12,783 (705) ^A^	2.7 (0.3) ^A^
4	13.3 (0.1) ^AB^	12,801 (707) ^A^	2.3 (0.4) ^A^
5	13.1 (0.2) ^A^	12,904 (340) ^A^	2.2 (0.4) ^A^
7	13.5 (0.1) ^B^	13,096 (794) ^A^	2.4 (0.2) ^A^
*p*-value	0.0282	0.5147	0.2324
R	0.63	0.21	−0.37
n	12	12	12

Standard deviations are given between brackets and greater than zero in all cases. For each cultivar, mean values in the same column differ significantly (*p* < 0.05, Tukey’s test) when they show different letters. The *p-*values, corresponding Pearson correlation coefficients (R) and number of observations (n) of the regression analysis are also reported.

**Table 8:** The crust-to-filling ratio, water evaporation during deep frying, expressed as the percentage of water present in potato mashes at the start of deep frying, and the firmness of the deep-fried potato mashes prepared from Fontane and Challenger potatoes stored for different times.

**Fontane**			
**Storage Month**	**Crust/Filling**	**Water Evaporation (%)**	**Firmness Filling (N/m^2^)**
2	0.37 (0.03) ^A^	49.8 (5.8) ^A^	919 (276) ^A^
4	0.37 (0.01) ^A^	50.5 (2.1) ^A^	1225 (226) ^A^
5	0.36 (0.01) ^A^	52.4 (1.1) ^A^	1145 (195) ^AB^
7	0.40 (0.02) ^A^	56.7 (3.1) ^A^	1153 (191) ^B^
8	0.39 (0.01) ^A^	58.2 (1.2) ^A^	1753 (194) ^C^
*p*-value	0.0747	0.0009	<0.0001
R	0.49	0.81	0.84
n	14	13	15
**Challenger**			
**Storage Month**	**Crust/Filling**	**Water Evaporation (%)**	**Firmness Filling (N/m^2^)**
2	0.34 (0.02) ^A^	36.7 (4.3) ^A^	1015 (264) ^A^
4	0.37 (0.02) ^A^	39.7 (1.3) ^A^	1471 (57) ^A^
5	0.34 (0.02) ^A^	37.2 (2.1) ^A^	1194 (223) ^A^
7	0.34 (0.01) ^A^	41.9 (1.5) ^A^	1276 (245) ^A^
*p*-value	0.9733	0.0681	0.3891
R	0.01	0.57	−0.27
n	12	12	12

Standard deviations are given between brackets and greater than zero in all cases. For each cultivar, mean values in the same column differ significantly (*p* < 0.05, Tukey’s test) when they show different letters. Means and standard deviations are given from 3 replicates, except for water evaporation for Fontane after 2 months of storage and the water evaporation and crust/filling ratio for Fontane after 5 months of storage, which were measured from 2 replicates. The *p-*values, corresponding Pearson correlation coefficients (R) and number of observations (n) of the regression analysis are also reported.

## Data Availability

The data that support the findings will be available in KU Leuven RDR at https://doi.org/10.48804/2HT1VB, following an embargo due to intellectual property rights until 30 September 2026.

## Supplementary Materials

The following supporting information can be downloaded at: https://www.mdpi.com/article/10.3390/foods15081433/s1, Supplementary Table S1: The degree of polymerization (DP) of amylose (AM) and amylopectin (AP) and the AM-to-AP ratio of the starches isolated from Fontane and Challenger potatoes as a function of storage time.; Supplementary Figure S1: Mechanical spectra of the storage (G′) and loss (G″) moduli of mash, prepared from Fontane potatoes that were stored for two months, as a function of oscillatory frequency (A) and as a function of strain (B). Supplementary Table S2: The area, T2 relaxation time (T2) and full width at half maximum (FWHM) of proton populations E and F in potato mashes prepared from Fontane and Challenger potatoes stored for different times. Supplementary Table S3: The oil content of the filling and crust and the resultant total oil content of the deep-fried potato mashes prepared from Fontane and Challenger potatoes stored for different times.

## Abbreviations

The following abbreviations are used in this manuscript:

AP	Amylopectin
AM	Amylose
CPMG	Carr−Purcell−Meiboom−Gill
T_C_	Conclusion temperature
DP	Degree of polymerization
EQPC	Diameter of a circle of equal projection area
DSC	Differential scanning calorimetry
DM	Dry matter
FID	Free induction decay
FWHM	Full width at half maximum
H	Enthalpy
T_c_-T_o_	Gelatinization temperature range
G″	Loss modulus
MC	Moisture content
N	Number of observations
OC	Oil content
T_O_	Onset temperature
T_p_	Peak temperature
R	Pearson correlation coefficient
G′	Storage modulus
TD ^1^H NMR	Time-domain proton nuclear magnetic resonance
