# Supplementary material for: The Impact of Post-Harvest Potato Storage on (Deep-Fried) Potato Mash Properties
Source: Foods. 2026 Apr 20;15(8):1433. doi: 10.3390/foods15081433 (PMC13114811; doi:10.3390/foods15081433)
Supplement: Supplementary file 1 [file foods-15-01433-s001.zip › foods-4198973-supplementary.pdf]

Supplementary Table S1. The degree of polymerization (DP) of amylose (AM) and amylopectin (AP) and the AM-to-AP ratio of the starches isolated from Fontane and Challenger potatoes as a function of storage time.

Fontane			
**Storage month**	**DP AM**	**DP AP**	**AM-to-AP**
**0**	931 (35)^{A}	23.5 (0.4)^{A}	0.36 (0.02)^{A}
**1**	968 (32)^{A}	23.5 (0.6)^{A}	0.36 (0.01)^{A}
**2**	952 (40)^{A}	23.5 (0.3)^{A}	0.36 (0.01)^{A}
**4**	961 (21)^{A}	23.5 (0.3)^{A}	0.36 (0.01)^{A}
**5**	953 (63)^{A}	23.7 (0.6)^{A}	0.36 (0.03)^{A}
**6**	945 (72)^{A}	23.9 (0.8)^{A}	0.36 (0.02)^{A}
**7**	968 (48)^{A}	23.7 (0.6)^{A}	0.37 (0.02)^{A}
**8**	946 (60)^{A}	23.5 (0.5)^{A}	0.36 (0.01)^{A}
***p*-value**	0.6881	0.3140	0.7195
**R**	0.05	0.11	0.04
**n**	80	80	80
**Challenger**			
**Storage month**	**DP AM**	**DP AP**	**AM-to-AP**
**0**	970 (57)^{AB}	23.6 (0.3)^{A}	0.37 (0.01)^{AB}
**1**	982 (52)^{AB}	23.4 (0.2)^{A}	0.36 (0.01)^{B}
**2**	968 (42)^{AB}	23.7 (0.5)^{A}	0.37 (0.01)^{AB}
**4**	1004 (40)^{A}	23.4 (0.1)^{A}	0.37 (0.01)^{AB}
**5**	913 (49)^{B}	23.6 (0.4)^{A}	0.37 (0.01)^{AB}
**6**	931 (39)^{B}	23.5 (0.3)^{A}	0.37 (0.01)^{AB}
**7**	973 (71)^{AB}	23.8 (0.7)^{A}	0.38 (0.01)^{A}
***p*-value**	0.1213	0.3465	0.0962
**R**	-0.19	0.11	0.20
**n**	70	70	70

Standard deviations are given between brackets. For each cultivar, mean values in the same column differ significantly ($p < 0.05$, Tukey's test) when they do not share the same letter. The *p*-values, corresponding Pearson correlation coefficients (R) and number of observations (n) of the regression analysis are also reported.

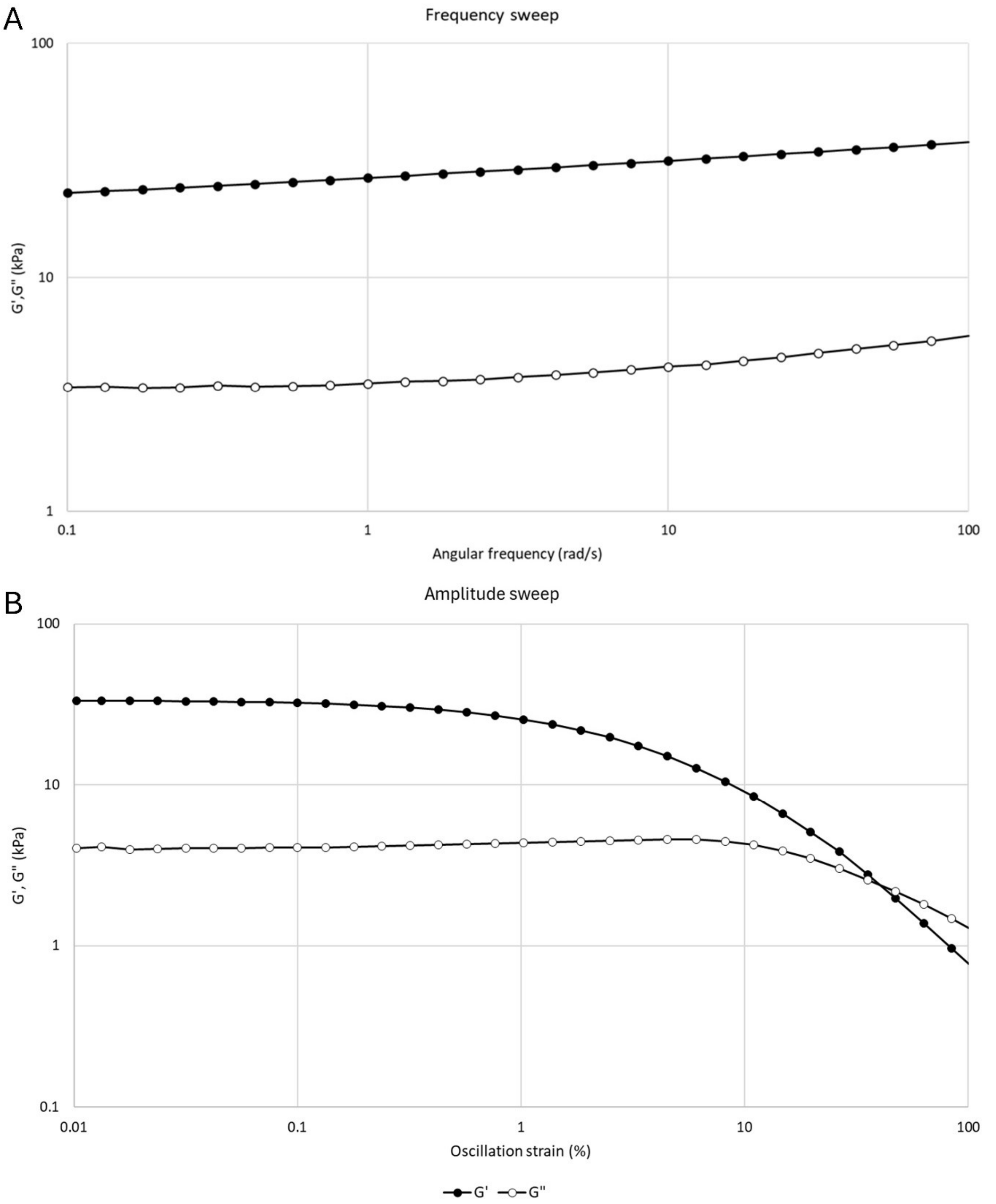

Supplementary Figure S1. Mechanical spectra of the storage (G') and loss (G") moduli of mash, prepared from Fontane potatoes that were stored for two months, as a function of oscillatory frequency (A) and as a function of strain (B).

Supplementary Table S2. The area, T_2 relaxation time (T_2) and full width at half maximum (FWHM) of proton populations E and F in potato mashes prepared from Fontane and Challenger potatoes stored for different times.

Storage month	T_2E (ms)	Area E/g (au)	FWHM E (ms)	T_2F (ms)	Area F/g (au)	FWHM F (ms)
**Fontane**						
**2**	34 (3)^{A}	677 (145)^{A}	13.8 (1.6)^{A}	180 (5)^{A}	14157 (2261)^{A}	90 (6)^{AB}
**4**	35 (2)^{A}	631 (53)^{A}	13.6 (1.3)^{A}	190 (7)^{A}	15790 (175)^{A}	90 (5)^{AB}
**5**	35 (2)^{A}	719 (35)^{A}	14.4 (0.5)^{A}	184 (5)^{A}	15340 (517)^{A}	94 (1)^{AB}
**7**	40 (14)^{A}	779 (29)^{A}	13.2 (0.7)^{A}	192 (26)^{A}	15486 (311)^{A}	97 (7)^{A}
**8**	36 (3)^{A}	825 (148)^{A}	12.1 (2.1)^{A}	190 (4)^{A}	15455 (106)^{A}	81 (3)^{B}
***p*-value**	0.4398	0.0198	0.1385	0.3379	0.2439	0.4633
**R**	0.22	0.61	-0.42	0.28	0.33	-0.21
**n**	14	14	14	14	14	14
**Challenger**						
**Storage month**	**T_2E (ms)**	**Area E/g (au)**	**FWHM E (ms)**	**T_2F (ms)**	**Area F/g (au)**	**FWHM F (ms)**
**2**	36 (4)^{A}	652 (74)^{A}	13.6 (0.4)^{AB}	195 (10)^{A}	15840 (90)^{A}	93.5 (8.6)^{AB}
**4**	42 (1)^{A}	600 (37)^{A}	13.8 (0.4)^{AB}	208 (10)^{A}	15853 (61)^{A}	95.0 (5.6)^{AB}
**5**	37 (2)^{A}	600 (10)^{A}	12.4 (0.9)^{A}	201 (4)^{A}	15901 (43)^{A}	88.4 (3.2)^{A}
**7**	43 (3)^{A}	719 (119)^{A}	16.3 (2.2)^{B}	206 (5)^{A}	15592 (63)^{B}	106.1 (7.6)^{B}
***p*-value**	0.0860	0.3399	0.0998	0.1804	0.0290	0.1283
**R**	0.52	0.30	0.50	0.41	-0.63	0.46
**n**	12	12	12	12	12	12

Standard deviations are given between brackets and greater than zero in all cases. For each cultivar, mean values in the same column differ significantly ($p < 0.05$, Tukey's test) when they do not share the same letter. The *p*-values, corresponding Pearson correlation coefficients (R) and number of observations (n) of the regression analysis are also reported.

Supplementary Table S3. The oil content of the filling and crust and the resultant total oil content of the deep-fried potato mashes prepared from Fontane and Challenger potatoes stored for different times.

Fontane oil content			
**Storage month**	**Filling (% on as is base)**	**Crust (% on as is base)**	**Total (% on as is base)**
**2**	1,8 (0,1)^{AB}	13,2 (0,9)^{A}	5,0 (0,2)^{AB}
**4**	1,9 (0,6)^{A}	14,5 (2,5)^{AB}	5,2 (1,2)^{A}
**5**	1,9 (0,3)^{A}	16,2 (0,3)^{AB}	5,6 (0,1)^{AB}
**7**	3,1 (1,8)^{AB}	17,5 (1,2)^{B}	7,2 (1,6)^{AB}
**8**	4,6 (0,3)^{B}	17,4 (0,6)^{AB}	8,1 (0,3)^{B}
***p*-value**	0.0030	0.0007	0.0010
**R**	0.73	0.79	0.80
**n**	14	14	14
**Challenger**			
**Storage month**	**Filling (% on as is base)**	**Crust (% on as is base)**	**Total (% on as is base)**
**2**	1,0 (0,2)^{A}	13,9 (0,9)^{A}	4,3 (0,5)^{A}
**4**	1,1 (0,1)^{A}	13,1 (1,9)^{A}	4,2 (0,5)^{A}
**5**	1,1 (0,4)^{A}	14,5 (0,7)^{A}	4,3 (0,2)^{A}
**7**	1,3 (0,2)^{A}	13,8 (1,6)^{A}	4,3 (0,5)^{A}
***p*-value**	0.1511	0.8429	0.8264
**R**	0.44	0.06	0.07
**n**	12	12	12

Standard deviations are given between brackets. For each cultivar, mean values in the same column differ significantly ($p < 0.05$, Tukey's test) when they do not share the same letter. The *p*-values, corresponding Pearson correlation coefficients (R) and number of observations (n) of the regression analysis are also reported.
